# Incidence and risk of herpes zoster in patients with ulcerative colitis and Crohn’s disease in the USA

**DOI:** 10.1093/gastro/goad016

**Published:** 2023-04-12

**Authors:** David Singer, Philippe Thompson-Leduc, Deepshekhar Gupta, Wendy Y Cheng, Sara Poston, Siyu Ma, John E Pawlowski, Mei Sheng Duh, Eric D Morris, Francesca Devine, Francis A Farraye

**Affiliations:** US Health Outcomes and Epidemiology—Vaccines, GSK, Philadelphia, PA, USA; Health Economics and Outcomes Research, Analysis Group, Inc., Montreal, Quebec, Canada; Health Economics and Outcomes Research, Analysis Group, Inc., Menlo Park, CA, USA; Health Economics and Outcomes Research, Analysis Group, Inc., Boston, MA, USA; US Health Outcomes and Epidemiology—Vaccines, GSK, Philadelphia, PA, USA; US Health Outcomes and Epidemiology—Vaccines, GSK, Philadelphia, PA, USA; Tufts Medical Center, Boston, MA, USA; Medical Affairs, GSK, Philadelphia, PA, USA; Health Economics and Outcomes Research, Analysis Group, Inc., Boston, MA, USA; Health Economics and Outcomes Research, Analysis Group, Inc., Menlo Park, CA, USA; Health Economics and Outcomes Research, Analysis Group, Inc., New York, NY, USA; Division of Gastroenterology and Hepatology, Mayo Clinic, Jacksonville, FL, USA

**Keywords:** ulcerative colitis, Crohn’s disease, herpes zoster

## Abstract

**Background:**

Patients with inflammatory bowel disease (IBD) are at increased risk of herpes zoster (HZ). We evaluated the incidence of HZ in ulcerative colitis (UC) and Crohn’s disease (CD) patients and compared this with HZ incidence in a non-IBD population.

**Methods:**

We conducted a retrospective cohort study (GSK study identifier: VEO-000043) of adults aged ≥18 years with UC and CD and without IBD who were identified from claims recorded in a US healthcare database between October 2015 and February 2020. Crude HZ incidence rates/1,000 person-years (PY) were calculated, and comparisons of HZ incidence rates between UC or CD and non-IBD cohorts were made using adjusted generalized linear models.

**Results:**

The study population comprised a total of 29,928 UC, 25,959 CD, and 11,839,329 non-IBD patients. Crude overall HZ incidence rates were 13.64/1,000 PY (UC), 15.94/1,000 PY (CD), and 7.95/1,000 PY (non-IBD). UC and CD patients had increased HZ incidence rates, with adjusted incidence rate ratios of 1.35 (95% confidence interval [CI], 1.26–1.44) and 1.66 (95% CI, 1.56–1.77), respectively, compared with non-IBD patients. Stratified analysis indicated increased relative rates of HZ in progressively younger age strata in the UC and CD patients compared with non-IBD patients. HZ incidence rates were higher in UC and CD patients who had previously received thiopurines or methotrexate, TNF-inhibitors, or corticosteroids than in UC and CD patients who did not take those medicines.

**Conclusion:**

UC and CD patients had increased HZ incidence rates compared with patients without IBD, demonstrating the importance of HZ prevention in IBD patients.

## Graphical Abstract

**Graphical Abstract:**
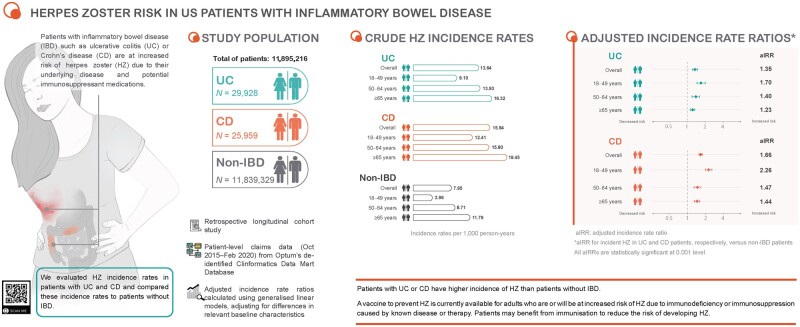

## Introduction

Herpes zoster (HZ) is an acute, painful, and debilitating condition that results from the reactivation of latent varicella–zoster virus (VZV) infection [1]. This reactivation is associated with an age-related decline in immunity (immunosenescence) and the risk of HZ is greater with increasing age [2]. In the USA, between 2007 and 2018, the estimated HZ incidence rate was 7.46 per 1,000 person-years (PY) in individuals aged 50–60 years, increasing to 12.01 per 1,000 PY in individuals aged >70 years [3]. HZ risk is higher in females [2, 3] and also greater in individuals with certain immunocompromising or autoimmune conditions, due to disease-associated immune dysfunction or the use of immunosuppressive therapies [4–6].

Studies evaluating HZ incidence in patients with inflammatory bowel disease (IBD) compared with HZ incidence in non-IBD or general populations have also reported a higher incidence of HZ in IBD patients [7–12], with a recent meta-analysis reporting a 1.68-fold increased risk of HZ in IBD patients [13]. There are conflicting data with regard to whether HZ risk is greater in patients with Crohn’s disease (CD) than in patients with ulcerative colitis (UC). While studies conducted in the UK, the USA, and South Korea have found a greater HZ risk in CD patients than that observed in UC cohorts [7, 8, 10, 11], more recent studies of US and Canadian populations have reported higher HZ incidence rates in UC patients [9, 14].

IBD medication use can also influence HZ risk. Most studies consistently reported a higher risk of HZ in IBD patients receiving systemic corticosteroids [7–11] and those receiving thiopurines [7–9]. Thiopurine use in combination with antitumour necrosis factor (TNF) agents has also been shown to be an independent risk factor for HZ in some studies [8, 9]. The evidence regarding the risk associated with anti-TNF agents as monotherapy is limited. A recent Korean study reported a greater risk of HZ in younger patients with UC and CD who were treated with anti-TNF agents [11]. However, a recent US study, predominantly involving patients >50 years of age, found no significantly increased risk of HZ with anti-TNF agents. A higher incidence of HZ has also been reported in IBD patients receiving Janus kinase (JAK) inhibitors, such as tofacitinib [15].

Previous studies in the USA evaluating HZ incidence in patients with IBD were conducted prior to more widespread use of anti-TNF agents and newer immunosuppressive agents [8] or chiefly evaluated HZ incidence in older adults [9]. There is a need for further data from a more recent, broader population of patients with IBD. The aim of the present study was to quantify HZ incidence in patients with UC and CD, and to compare this incidence with that observed in a broader population of patients without IBD, using data from a large US healthcare claims database.

## Materials and methods

### Study design and data sources

This was a retrospective longitudinal cohort study (GSK study identifier: VEO-000043) using patient-level medical and pharmacy claims data sourced from the Optum’s de-identified Clinformatics Data Mart Database. This contains healthcare information on 15–19 million annual covered lives insured by commercial or Medicare Advantage plans across all 50 states in the USA. The database captures enrolment, demographics, health plan eligibility, and medical and prescription-drug claims arising from inpatient stays and outpatient care, identifiable via diagnostic and medication codes.

**Figure 1.**
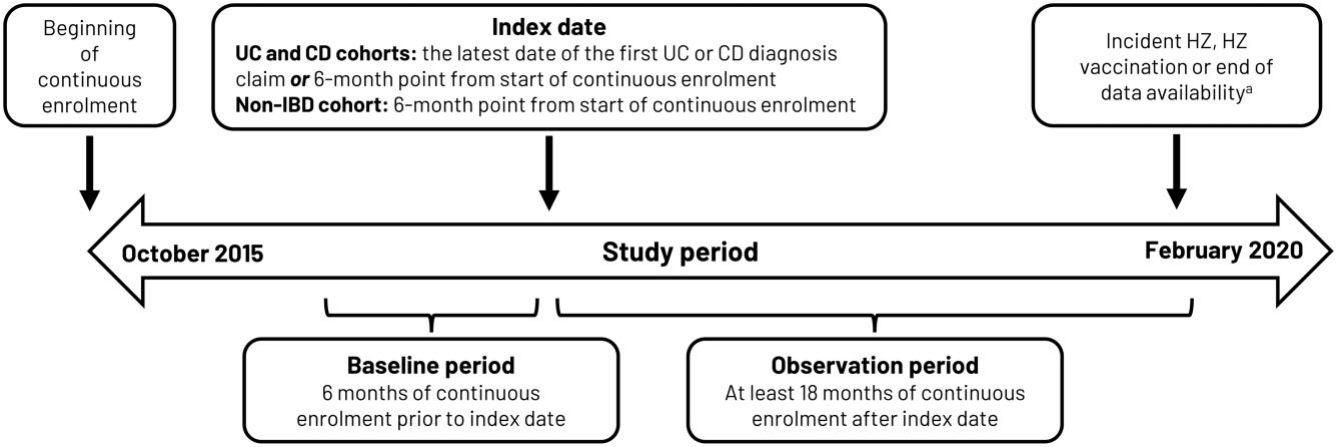
Study design schematic. ^a^All patients were followed up for ≥18 months post-index date, or until the incident HZ (event) or HZ vaccination (censor). Patients with neither HZ nor HZ vaccination were censored at the end of their continuous enrolment period. CD, Crohn’s disease; HZ, herpes zoster; UC, ulcerative colitis.

The study utilized data spanning the period from 1 October 2015 to 28 February 2020 to estimate HZ incidence rates in UC and CD patients, evaluated as separate and mutually exclusive cohorts, with each compared with a single non-IBD patient cohort (Figure 1).

### Study population

Eligible individuals were patients ≥18 years of age with ≥6 months of continuous enrolment prior to the index date (which marked the beginning of observation for incident HZ) and ≥18 months of continuous enrolment after the index date. Patients were assigned to one of three mutually exclusive study cohorts (UC, CD, and non-IBD cohorts) on the basis of claims associated with relevant International Classification of Diseases, 10th Revision, Clinical Modification (ICD-10-CM) codes. For the UC cohort, patients were required to have at least two claims associated with a UC diagnosis (ICD-10-CM, K51) on separate days within a 12-month period, or at least one claim associated with a UC diagnosis and at least one pharmacy claim for an IBD-related medication (e.g. aminosalicylic acids, mesalamine, olsalazine, balsalazide, sulfasalazine, 6-mercaptopurine, azathioprine, thiopurine, enteral budesonide, biologics, tofacitinib) within 30 days after the UC diagnosis. The index date was the date of the first UC diagnosis claim or the 6-month point from the start of continuous enrolment, whichever was latest. CD cohort inclusion followed a similar approach, with patients identified using ICD-10-CM diagnosis code K50 (Figure 1). For patients with claims for both UC and CD, a majority-based algorithm approach was used to allocate patients to the UC or CD cohorts, as used in previous studies [16, 17]. For the non-IBD cohort, patients were required to have had no claim associated with a UC or CD diagnosis at any time, with the index date being the 6-month point from the start of continuous enrolment. For each cohort, patients were excluded if they had any claims associated with an HZ diagnosis (including HZ complications) prior to or on the index date (identified via ICD-10-CM codes B02.0–B02.9). Patients with prior receipt of HZ vaccination (identified by Current Procedural Terminology [CPT], National Drug Code [NDC], and Generic Product Identifier codes) were also excluded. The codes used to identify all relevant medications or HZ vaccines are shown in Supplementary Table 1.

### Study data and outcomes

Baseline patient demographics, i.e. age, sex, region, and insurance plan type, were identified as of the index date. Clinical characteristics were assessed during the 6-month baseline period prior to the index date. For all cohorts this included an assessment of co-morbidities, conditions associated with a potentially increased risk of HZ, and immunosuppressive medication use (identified via relevant ICD-10-CM and medication codes). Overall co-morbidity burden was estimated using the modified Charlson–Quan co-morbidity index (CCI) [18]. Markers of IBD severity (weight loss, malnutrition, anaemia) and clinical management details (including surgeries and procedures) were also assessed during the baseline period. The use of IBD-related medications was captured during the baseline period and throughout the observation period. Specific medications/medication groups were categorized utilizing a laddered approach into mutually exclusive (and collectively exhaustive) groups as (i) no therapy, 5-aminosalicylate (ASA), or budesonide; (ii) thiopurine or methotrexate; (iii) vedolizumab and/or ustekinumab; (iv) TNF-inhibitors (adalimumab, certolizumab, or infliximab); (v) any biologic in combination with methotrexate or thiopurine; (vi) JAK inhibitors; and (vii) corticosteroids (other than budesonide).

All patients were followed up for ≥18 months post-index date or until incident HZ (event) or HZ vaccination (censor). Patients with neither HZ nor HZ vaccination were censored at the end of their continuous enrolment period. Incident HZ was defined as at least one claim associated with an HZ diagnosis (ICD-10-CM codes B02.0–B02.9, with the exception of B02.2) occurring during the observation period.

The study objectives were to (i) estimate crude and age-stratified HZ incidence rates per 1,000 PY in the UC, CD, and non-IBD cohorts, and (ii) compare incidence rates (adjusted for baseline clinical and demographic characteristics) in patients with UC or with CD vs those patients without IBD. For the UC and CD cohorts, an additional objective was to estimate HZ incidence rates in patients receiving specific IBD medications.

### Statistical analyses

Descriptive analyses were performed for all study variables for each cohort. For continuous variables, mean values ± standard deviations (SD) and medians were calculated, while frequency counts and percentages were calculated for categorical variables. The balance of baseline characteristics across cohorts was assessed using standardized differences, where standardized differences of 20%, 50%, and 80% suggest small, medium, and large differences between cohorts, respectively [19].

HZ incidence rates were calculated as the number of patients with incident HZ divided by the person-time observed and expressed per 1,000 PY. Incidence was calculated for each cohort (UC, CD, and non-IBD), both overall and stratified by age. Adjusted incidence rate ratios (aIRRs) were estimated to compare HZ incidence rates between UC and non-IBD cohorts, and between CD and non-IBD cohorts, using generalized linear models with a Poisson distribution, and log link, and accounting for differences in patients’ baseline characteristics using doubly-robust propensity score adjustment. In this approach, propensity scores were estimated using logistic regression with IBD status as the outcome, i.e. UC vs non-IBD and, separately, CD vs non-IBD. Key baseline demographics (year of index date, age, sex, geographic region, and insurance type) and clinical variables (e.g. CCI scores, any co-morbidity potentially associated with HZ, additional immunosuppressive conditions) were included in the propensity score models as predictors (Supplementary Table 2). In this approach, each predictor variable and also the overall propensity scores were included in the final generalized linear models.

### Ethics approval and consent to participate

The study employed a retrospective cohort study design using a large administrative claims database. Data were de-identified and comply with the requirements of the Health Insurance Portability and Accountability Act. Institutional Review Board review and approval were therefore not required, as per the United States Department of Health and Human Services regulation for the protection of human subjects in research (45 CFR 46) [20]. The study has been conducted in accordance with the guiding principles of the Declaration of Helsinki. As only existing de-identified data have been analysed and as patients have not been contacted during the course of this study, the informed consent process is not applicable.

## Results

### Study population and baseline characteristics

**Figure 2.**
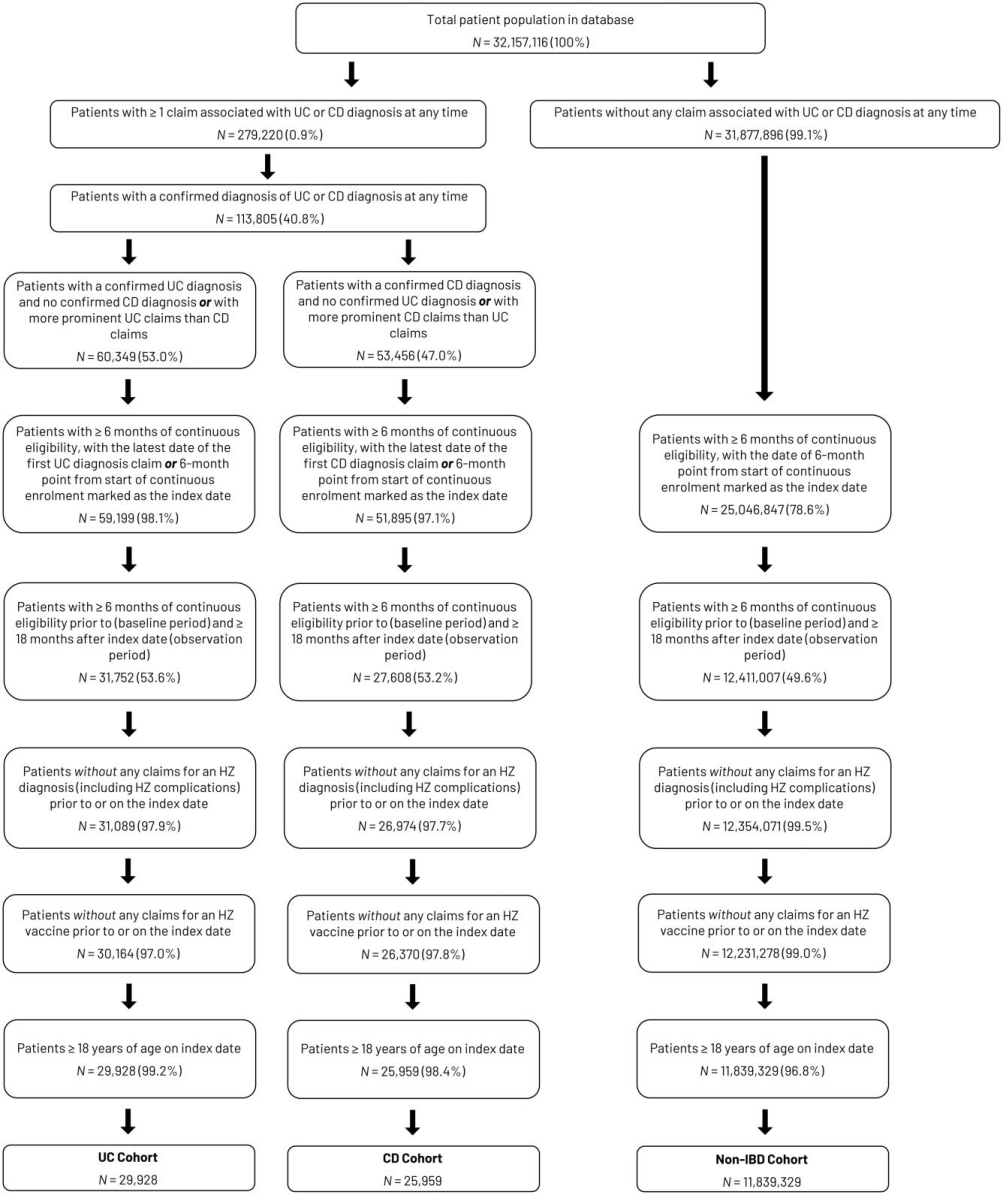
Patient selection and assignment in study cohorts. CD, Crohn’s disease; HZ, herpes zoster; IBD, inflammatory bowel disease; *N*, number of patients; UC, ulcerative colitis.

Between 1 October 2015 and 28 February 2020, the database included 32,157,116 patients, of whom 279,220 (0.9%) had at least one claim for IBD (either UC or CD) at any time and 113,805 had a confirmed UC or CD diagnosis. After applying the eligibility criteria of the study, the final cohorts included 29,928 patients with UC, 25,959 patients with CD, and 11,839,329 patients without IBD (Figure 2). Selected baseline demographic and clinical characteristics at, or in the 6 months prior to, the index date for each cohort are shown in Table 1.

**Table 1.** Baseline demographics and clinical characteristics of the study cohorts^a^

Demographic and clinical characteristic	UC (*N *=* *29,928)	Non-IBD (*N *=* *11,839,329)	Standardized difference^b^	CD (*N *=* *25,959)	Non-IBD (*N *=* *11,839,329)	Standardized difference^b^
Year of index date, *n* (%)						
2016	1,737 (5.8)	8,187,483 (69.2)	130.9%	2,873 (11.1)	8,187,483 (69.2)	118.5%
2017	17,333 (57.9)	2,215,591 (18.7)	80.6%	15,337 (59.1)	2,215,591 (18.7)	82.8%
2018	10,858 (36.3)	1,436,255 (12.1)	56.4%	7,749 (29.9)	1,436,255 (12.1)	43.5%
Age at index date, years, mean ± SD	60.0 ± 17.4	54.0 ± 19.0	33.1%	56.2 ± 17.9	54.0 ± 19.0	11.7%
18–29, *n* (%)	1,851 (6.2)	1,622,340 (13.7)	25.1%	2,622 (10.1)	1,622,340 (13.7)	11.1%
30–39, *n* (%)	3,133 (10.5)	1,642,654 (13.9)	10.4%	3,091 (11.9)	1,642,654 (13.9)	5.9%
40–49, *n* (%)	3,635 (12.1)	1,648,014 (13.9)	5.3%	3,665 (14.1)	1,648,014 (13.9)	0.6%
18–49, *n* (%)	8,619 (28.8)	4,913,008 (41.5)	26.6%	9,378 (36.1)	4,913,008 (41.5)	11.0%
50–64, *n* (%)	6,684 (22.3)	2,541,927 (21.5)	2.1%	6,444 (24.8)	2,541,927 (21.5)	8.0%
≥65, *n* (%)	14,625 (48.9)	4,384,394 (37.0)	23.9%	10,137 (39.1)	4,384,394 (37.0)	4.2%
Female, *n* (%)	16,195 (54.1)	6,172,003 (52.1)	4.0%	14,770 (56.9)	6,172,003 (52.1)	9.6%
Geographic region, *n* (%)						
South	12,076 (40.4)	4,827,240 (40.8)	0.9%	10,800 (41.6)	4,827,240 (40.8)	1.7%
West	6,433 (21.5)	2,712,093 (22.9)	3.4%	4,828 (18.6)	2,712,093 (22.9)	10.6%
Midwest	7,075 (23.6)	2,752,174 (23.2)	0.9%	6,891 (26.5)	2,752,174 (23.2)	7.6%
Northeast	4,306 (14.4)	1,307,321 (11.0)	10.0%	3,413 (13.1)	1,307,321 (11.0)	6.5%
Unknown	38 (0.1)	240,501 (2.0)	18.4%	27 (0.1)	240,501 (2.0)	18.8%
Insurance type, *n* (%)						
Medicare advantage	15,486 (51.7)	4,665,584 (39.4)	24.8%	12,435 (47.9)	4,665,584 (39.4)	17.1%
Commercial	14,442 (48.3)	7,173,745 (60.6)	24.8%	13,524 (52.1)	7,173,745 (60.6)	17.1%
CCI, mean ± SD (median)	0.8 ± 1.4 (0.0)	0.4 ± 1.0 (0.0)	31.7%	0.8 ± 1.4 (0.0)	0.4 ± 1.0 (0.0)	29.6%
0, *n* (%)	19,435 (64.9)	9,466,939 (80.0)	33.6%	16,943 (65.3)	9,466,939 (80.0)	32.9%
1, *n* (%)	4,144 (13.8)	1,077,636 (9.1)	14.9%	3,713 (14.3)	1,077,636 (9.1)	16.2%
2–4, *n* (%)	5,384 (18.0)	1,155,967 (9.8)	23.8%	4,592 (17.7)	1,155,967 (9.8)	23.0%
≥5, *n* (%)	965 (3.2)	138,787 (1.2)	14.0%	711 (2.7)	138,787 (1.2)	11.3%
Co-morbidities potentially associated with HZ, *n* (%)	2,048 (6.8)	317,280 (2.7)	19.6%	2,097 (8.1)	317,280 (2.7)	23.9%
Use of IBD-related medications, *n* (%)	14,936 (49.9)	396,090 (3.3)	105.3%	13,847 (53.3)	396,090 (3.3)	110.9%
Aminosalicylate (5-ASA)	10,556 (35.3)	8,890 (0.1)	92.3%	4,517 (17.4)	8,890 (0.1)	61.4%
Methotrexate	366 (1.2)	44,494 (0.4)	9.5%	576 (2.2)	44,494 (0.4)	16.3%
Thiopurines	2,084 (7.0)	6,900 (0.1)	37.5%	3,002 (11.6)	6,900 (0.1)	49.2%
Ustekinumab	34 (0.1)	3,019 (0.0)	3.3%	328 (1.3)	3,019 (0.0)	15.5%
Vedolizumab	592 (2.0)	3 (0.0)	20.0%	768 (3.0)	3 (0.0)	24.5%
Anti-TNF biologics	2,435 (8.1)	19,182 (0.2)	40.0%	5,652 (21.8)	19,182 (0.2)	69.2%
JAK inhibitors	17 (0.1)	1,518 (0.0)	2.4%	8 (0.0)	1,518 (0.0)	1.2%
Systemic steroids	3,418 (11.4)	340,251 (2.9)	33.2%	3,402 (13.1)	340,251 (2.9)	37.7%

Patient demographic characteristics were identified as of the index date. Clinical characteristics were assessed during the 6-month period strictly prior to the index date.

a A more complete version of this table is available in the Supplementary Material (see Supplementary Table 3).

b Standardized differences of 20%, 50%, and 80% suggest small, medium, and large differences between cohorts, respectively [19].

5-ASA, 5-aminosalicylate; AIDS, acquired immunodeficiency syndrome; CCI, modified Charlson–Quan co-morbidity index; CD, Crohn’s disease; HIV, human immunodeficiency virus; HZ, herpes zoster; IBD, inflammatory bowel disease; JAK, Janus kinase; *N*, number of patients in the cohort; *n*, number of patients in a category; SD, standard deviation; TNF, tumour necrosis factor; UC, ulcerative colitis.

The majority of patients in the UC and CD cohorts had an index date in 2017 (57.9% and 59.1%, respectively) or 2018 (36.3% and 29.9%, respectively), whereas 69.2% of the non-IBD cohort had a 2016 index date. Of the UC cohort, 54.1% were female, with a mean age (± SD) of 60.0 ± 17.4 years. For the CD cohort, 56.9% were female, with a mean age of 56.2 ± 17.9 years; for the non-IBD cohort, 52.1% were female, with a mean age of 54.0 ± 19.0 years. The co-morbidity burden was similar in the UC and CD cohorts (mean CCI 0.8 ± 1.4) and greater than that observed in the non-IBD cohort (0.4 ± 1.0). The prevalence of specific co-morbidities varied between cohorts, although for most co-morbidities, standardized differences were <20% (Supplementary Table 3). Propensity score histograms for the full UC, CD, and non-IBD cohorts are shown in Supplementary Figure 1.

Baseline use of IBD-related medications (as assessed during the 6-month period prior to the index date) was common, with nearly half of the patients in the UC cohort (49.9%) and slightly more in the CD cohort (53.3%) using IBD-related medications. The use of specific classes of medications was slightly lower in the UC cohort than in the CD cohort: thiopurines (7.0% and 11.6%, respectively), anti-TNF biologics (8.1% and 21.8%, respectively), and corticosteroid use (11.4% and 13.1%, respectively) (Supplementary Figure 2). Relatively few patients in either cohort were receiving ustekinumab or vedolizumab. In the non-IBD cohort, the use of IBD-related medications for other co-morbidities was low (3.3%), although corticosteroid use was reported in 2.9% of patients at baseline.

### HZ incidence in UC, CD, and non-IBD cohorts

**Figure 3.**
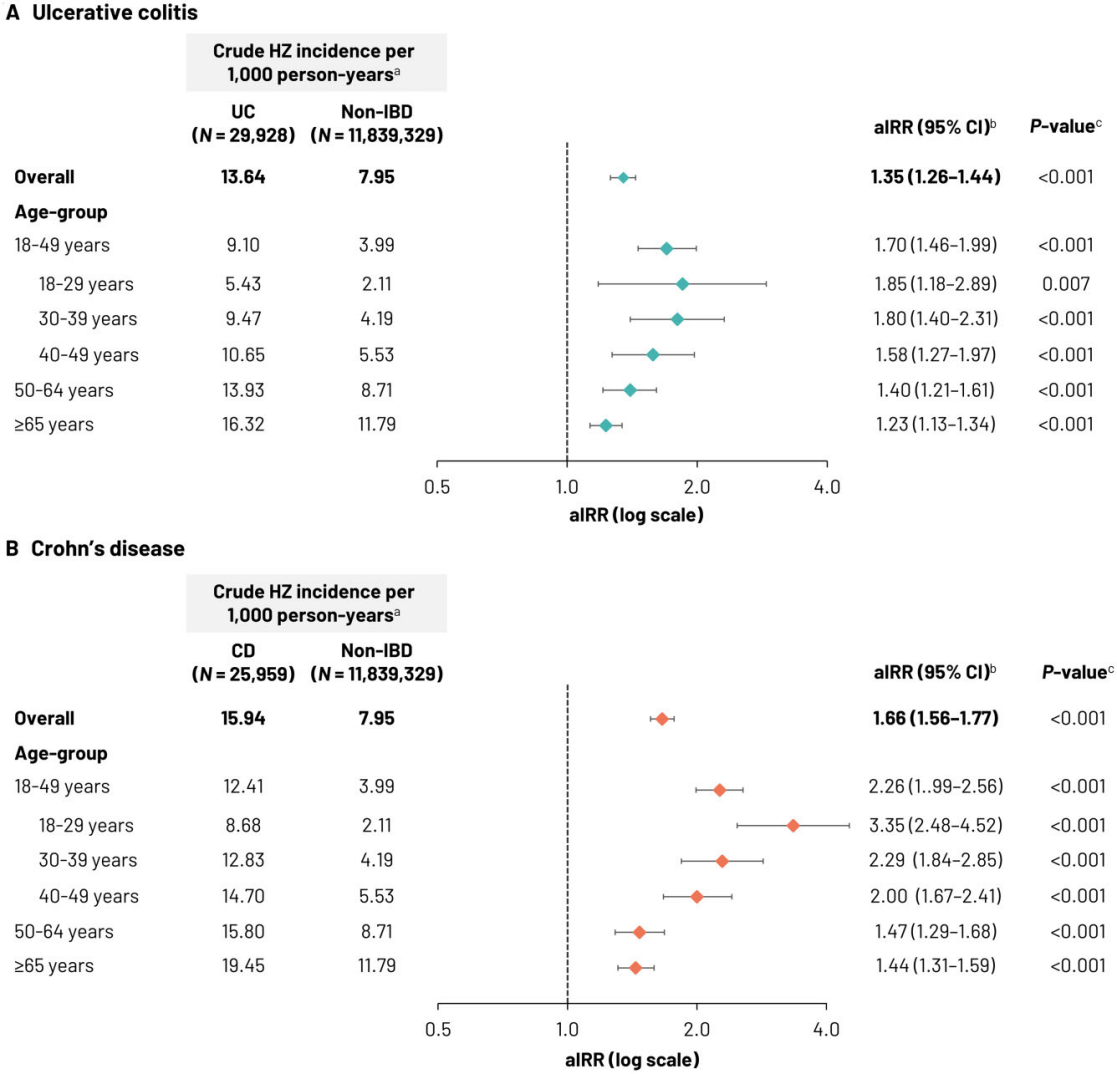
Crude HZ incidence rates and adjusted incidence rate ratios in the (A) UC and (B) CD cohorts compared with the non-IBD cohort. ^a^Incidence rates were calculated as the number of patients with incident HZ divided by the person-time observed expressed per 1,000 person-years. ^b^Adjusted incidence rate ratios (aIRRs) were calculated using generalized linear models with a Poisson distribution, adjusting for patients’ propensity score and relevant baseline characteristics/baseline covariates. ^c^*P*-values for aIRRs were calculated using the Poisson distribution. aIRR, adjusted incidence rate ratio; CD, Crohn’s disease; CI, confidence interval; HZ, herpes zoster; IBD, inflammatory bowel disease; *N*, number of patients in the cohort; SD, standard deviation; UC, ulcerative colitis.

Patients with UC were followed up for a mean time (± SD) of 25.3 ± 7.0 months and those with CD were followed up for 26.4 ± 7.6 months; the non-IBD cohort was followed up for 34.3 ± 11.2 months. The study included a total of 33,939,920 PY of observation time, with 62,986 PY for the UC cohort, 57,141 PY for the CD cohort, and 33,819,793 PY for the non-IBD cohort. A total of 270,629 cases of incident HZ were identified with 859 in the UC cohort, 911 in the CD cohort, and 268,859 HZ cases in the non-IBD cohort. The overall crude incidence rate for HZ was 13.64 per 1,000 PY in the UC cohort, 15.94 per 1,000 PY in the CD cohort, and 7.95 per 1,000 PY in the non-IBD cohort (Table 2 and Figure 3). In all three cohorts, the HZ incidence rate increased with increasing age. In patients aged 18–49 years, the HZ incidence rates were 9.10 per 1,000 PY in patients with UC and 12.41 per 1,000 PY in patients with CD, compared with an HZ incidence rate of 3.99 per 1,000 PY in patients without IBD. In patients aged ≥65 years, the corresponding HZ incidence rates were 16.32, 19.45, and 11.79 per 1,000 PY for the UC, CD, and non-IBD cohorts, respectively.

**Table 2.** Crude HZ incidence rates per 1,000 person-years in UC, CD, and non-IBD cohorts

Age group	Variable	UC	CD	Non-IBD
Overall	*N*	29,928	25,959	11,839,329
	HZ cases	859	911	268,859
	Observation-years^a^	62,986	57,141	33,819,793
	Incidence rate^b^	13.64	15.94	7.95
18–29 years	*N*	1,851	2,622	1,622,340
	HZ cases	22	51	9,352
	Observation-years^a^	4,048	5,877	4,429,353
	Incidence rate^b^	5.43	8.68	2.11
30–39 years	*N*	3,133	3,091	1,642,654
	HZ cases	65	89	19,387
	Observation-years^a^	6,863	6,939	4,630,149
	Incidence rate^b^	9.47	12.83	4.19
40–49 years	*N*	3,635	3,665	1,648,014
	HZ cases	84	122	26,391
	Observation-years^a^	7,888	8,302	4,768,209
	Incidence rate^b^	10.65	14.70	5.53
All 18–49 years	*N*	8,619	9,378	4,913,008
	HZ cases	171	262	55,130
	Observation-years^a^	18,799	21,118	13,827,711
	Incidence rate^b^	9.10	12.41	3.99
50–64 years	*N*	6,684	6,444	2,541,927
	HZ cases	193	223	62,219
	Observation-years^a^	13,858	14,117	7,145,465
	Incidence rate^b^	13.93	15.80	8.71
≥65 years	*N*	14,625	10,137	4,384,394
	HZ cases	495	426	151,510
	Observation-years^a^	30,329	21,906	12,846,617
	Incidence rate^b^	16.32	19.45	11.79

a Follow-up for observation for incident HZ.

b Incidence rates calculated as the number of patients with incident HZ divided by the person-time observed expressed per 1,000 person-years.

CD, Crohn’s disease; HZ, herpes zoster; IBD, inflammatory bowel disease; *N*, number of patients in a cohort or relevant age-stratum; SD, standard deviation; UC, ulcerative colitis.

After adjustment for baseline covariates, compared with non-IBD patients, the aIRR for incident HZ in UC patients was 1.35 (95% confidence interval [CI], 1.26–1.44; *P *<* *0.001). Age-specific aIRRs were higher in UC patients aged <50 years, with an aIRR of 1.85 (95% CI, 1.18–2.89; *P *=* *0.007) in patients aged 18–29 years and 1.80 (95% CI, 1.40–2.31; *P *<* *0.001) in those aged 30–39 years (Figure 3; unadjusted IRRs are shown in Supplementary Table 4). For patients with CD vs non-IBD patients, the aIRR for HZ was 1.66 (95% CI, 1.56–1.77; *P *<* *0.001). aIRRs were higher still in younger patients, with aIRRs of 3.35 (95% CI, 2.48–4.52) and 2.29 (95% CI, 1.84–2.85), respectively, in patients aged 18–29 and 30–39 years (both *P *<* *0.001).

### HZ incidence associated with IBD medication use

In both IBD cohorts, patients receiving corticosteroids had the highest HZ incidence rates: 24.07 per 1,000 PY in the UC cohort and 28.64 per 1,000 PY in the CD cohort (Table 3 and Supplementary Table 5). HZ incidence rates were also high in those patients with prior receipt of thiopurines or methotrexate: 18.50 per 1,000 PY in the UC cohort and 28.20 per 1,000 PY in the CD cohort.

**Table 3.** Crude HZ incidence rates per 1,000 person-years in UC and CD cohorts receiving specific IBD medications

Medication category	Variable	UC	CD
Any	*N*	29,928	25,959
	HZ cases	859	911
	Observation-years^a^	62,986	57,141
	Incidence rate^b^	13.64	15.94
No therapy, 5-ASA, or budesonide^c^	*N*	22,430	14,919
	HZ cases	570	415
	Observation-years^a^	46,837	32,437
	Incidence rate^b^	12.17	12.79
Thiopurine or methotrexate^c^	*N*	1,385	1,629
	HZ cases	54	98
	Observation-years^a^	2,919	3,475
	Incidence rate^b^	18.50	28.20
Vedolizumab and/or ustekinumab^c^	*N*	985	1,980
	HZ cases	22	54
	Observation-years^a^	2,258	4,584
	Incidence rate^b^	9.74	11.78
Adalimumab, certolizumab, or infliximab^c^	*N*	1,861	4,080
	HZ cases	59	141
	Observation-years^a^	4,099	9,189
	Incidence rate^b^	14.39	15.34
Any biologic in combination with methotrexate or thiopurine^c^	*N*	411	768
	HZ cases	14	39
	Observation-years^a^	889	1,711
	Incidence rate^b^	15.75	22.80
JAK inhibitors^c^	*N*	148	23
	HZ cases	4	1
	Observation-years^a^	334	55
	Incidence rate^b^	11.99	18.17
Corticosteroid other than budesonide, alone or in combination with the medications above^c^	*N*	2,708	2,560
	HZ cases	136	163
	Observation-years^a^	5,651	5,691
	Incidence rate^b^	24.07	28.64

a Follow-up for observation for incident HZ.

b Incidence rates calculated as the number of patients with incident HZ divided by the person-time observed expressed per 1,000 person-years.

c Specific medications/medication groups were categorized utilizing a laddered approach into mutually exclusive (and collectively exhaustive) groups as (i) no therapy, 5-ASA, or budesonide; (ii) thiopurine or methotrexate; (iii) vedolizumab and/or ustekinumab; (iv) TNF-inhibitors (adalimumab, certolizumab, or infliximab); (v) any biologic in combination with methotrexate or thiopurine; (vi) JAK inhibitors; and (vii) corticosteroids (other than budesonide).

5-ASA, 5-aminosalicylate; CD, Crohn’s disease; HZ, herpes zoster; IBD, inflammatory bowel disease; JAK, Janus kinase; *N*, number of patients in a cohort or relevant medication category; SD, standard deviation; TNF, tumour necrosis factor; UC, ulcerative colitis.

## Discussion

In this retrospective claims-database analysis, we evaluated HZ incidence rates in patients with IBD, stratified into mutually exclusive UC and CD patient cohorts, and evaluated the risk of HZ compared with a large, non-IBD population using adjusted multivariate analyses. We found that the HZ incidence rate was higher in both UC and CD patients (13.64 and 15.94 per 1,000 PY, respectively) than in the patients without IBD (7.95 per 1,000 PY). UC and CD were associated with an increased risk of HZ, with a 1.35-fold greater incidence of HZ in UC patients and a 1.66-fold greater incidence of HZ in CD patients than in the non-IBD population, after adjusting for baseline differences between cohorts. These findings are consistent with previous studies reporting a higher HZ incidence rate and greater relative risk of HZ in IBD patients [7–14, 21]. We also found that crude HZ incidence rate was numerically higher in patients with CD than in UC patients, although this was not formally compared in our study. This is again consistent with some previous studies from the UK, the USA, and South Korea, which found a greater risk of HZ in CD patients than that observed in UC patients [7, 8, 10, 11]. However, other studies have reported a greater incidence of HZ in UC patients. A recent large study by Khan *et al*. in the USA found a slightly higher HZ risk in UC patients than in patients with CD, with adjusted hazard ratios (HRs) of 1.81 (95% CI, 1.56–2.11) for UC patients and 1.56 (95% CI, 1.28–1.91) for CD patients compared with a non-IBD cohort, although the risk differences between the UC and CD cohorts were not significant [9]. However, it is worth noting that in the present study, differences in HZ incidence rates in the UC and CD cohorts may have been influenced by medication use. When looking only at those patients receiving conservative treatment (no therapy, 5-ASA, or budesonide), the HZ incidence rate was broadly comparable in the UC and CD cohorts (12.17 and 12.49 per 1,000 PY, respectively).

In previous studies [2, 3], the HZ incidence rate was highest in older patients and this was seen in the present study within all cohorts, consistently with the role played by immunosenescence in VZV reactivation. However, the HZ incidence rate was also relatively high in younger UC and CD patients (<50 years of age) and the relative incidence rate of HZ was greatest in younger patients when compared between IBD and non-IBD cohorts. For example, the greatest adjusted increase in the HZ incidence rate was observed in CD patients aged 18–29 years with a 3.3-fold greater risk of HZ compared with non-IBD patients in this age group. Similar findings of increased relative risk in progressively younger age strata have also been reported in South Korean patients with UC or CD [11]. Furthermore, similar observations of greater HZ relative risk in younger patients have been reported for other immune-mediated conditions (e.g. rheumatoid arthritis and systemic lupus erythematosus) [4, 22].

We also evaluated the HZ incidence associated with IBD medication use. HZ incidence rates were lower among patients receiving conservative treatment (no therapy, 5-ASA, or budesonide) than among patients receiving other therapies—a pattern seen in both the UC and CD cohorts. HZ incidence rates were higher in patients receiving TNF-inhibitors, even higher in those receiving thiopurines or methotrexate, and highest in those also receiving corticosteroids. In contrast to some previous US studies that evaluated HZ risk associated with medication exposure time or compared HZ risk in IBD patients receiving specific medications relative to those IBD patients receiving only 5-ASA [8, 9], our study focused on describing the incidence rates of HZ among patients with UC and CD stratified by medication at the time they had an HZ event or were censored. Such differences in study design and analytical approach make direct comparisons with previously reported estimates of risk associated with IBD medication in UC and CD patients challenging. However, our overall findings of higher HZ incidence rates in UC and CD patients receiving thiopurines or methotrexate and those receiving corticosteroids are consistent with previous studies in IBD patients that report greater risk for HZ associated with thiopurines [7–9] and corticosteroids [7–11].

This study has several limitations. It was conducted using administrative claims data and thus has limitations common to all such retrospective database analyses, such as billing record miscoding, missing data, and a lack of detailed clinical data. While we used ICD-10-CM codes to identify patients with HZ, this may have failed to identify some eligible patients, including those patients with less severe HZ who did not seek medical care. As such, HZ case numbers and the resultant incidence estimations may be underestimates. It is also possible that some patients identified as having HZ were misdiagnosed based on the presentation of disease, which could have resulted in misclassification in the study. While we adjusted for potential confounders using measures of patients’ baseline clinical and demographic characteristics using a doubly-robust approach (by including both propensity score and baseline variables in our models for comparisons of incidence rates), residual confounding may have influenced our findings. For example, studies from Taiwan have found that HZ risk is higher in patients following appendectomy or splenectomy, and with other co-morbidities such as varicocele [23–25]. While such associations have not yet been investigated in US populations (and were not considered in previous studies of HZ risk in US IBD populations [8, 9]), we accept that these remain potential confounders (although the impact may have been rather limited).

Our pragmatic approach to medication groupings has some limitations. For example, we grouped patients receiving no therapy or receiving budesonide or 5-ASA as a single category, where our goal was to look at those patients receiving no or only limited immunosuppressive therapy. However, 5-ASA may carry some additive risk for HZ, as reported in previous US studies, e.g. by Long *et al.*, although any such risk was non-significant [8], and more recently Khan *et al.*, who found a significantly higher risk in both UC and CD patients receiving 5-ASA than in non-IBD patients [9].

The study included a relatively high number of patients in our UC and CD cohorts characterized as receiving no IBD medications during the 6-month period prior to the index date (50% of UC patients and 47% of CD patients). While this proportion of patients is relatively large, previous US studies using insurance claims data have reported similarly large proportions of IBD patients receiving no therapy [8, 26]. While this may reflect low disease activity in these patients, our analyses (common to many such studies utilizing insurance claims data) were hampered by a lack of available clinical information on disease activity or status (e.g. in remission, etc.). Our grouping of these patients along with those receiving 5-ASA or budesonide hinders our ability to fully assess any baseline impact of IBD in the absence of treatment on HZ incidence. However, our overall findings of higher HZ incidence rates and higher adjusted HZ risk estimates across all-age strata (irrespective of medications received) and the higher incidence rates in patients receiving conservative therapies in the ‘no therapy, 5-ASA, or budesonide’ category (many of whom were on no therapy) would suggest that both UC and CD impart a higher risk for HZ.

We grouped vedolizumab and ustekinumab together, in part to distinguish these agents from other medication classes with recognized HZ risk (e.g. JAK inhibitors). However, vedolizumab and ustekinumab have different mechanisms of actions, and may also differ in their potential impact on risk for HZ. In the present study, our findings that the crude HZ incidence rates in our UC and CD cohorts for patients receiving vedolizumab and/or ustekinumab were lower than that observed for the ‘no therapy or receiving budesonide or receiving 5-ASA’ category would be consistent with previous data indicating little additive risk [27–29] and may further reflect the type of patients receiving these treatments.

A further limitation of our medication analyses was in only addressing medication use at the end of the observation period for incident HZ, and we did not account for exposure to other medications preceding this, or for therapy switching or medication adherence.

A key strength of the present study is that our analysis was conducted across a large adult population that included a high proportion of patients <50 years of age (UC, 29% and CD, 36%) and with a slight majority of female patients in each cohort (ranging from 52% to 57%). In this respect, our study is complementary to other recent US studies, which focused on predominantly male (>90%) and slightly older populations [9, 30]. In addition, our population is relatively recent (from 2016 to 2020) and can provide a more recent perspective than previous US studies [8, 9].

The higher HZ incidence rates and increased risk of HZ in UC and CD patients that we reported may provide support for the broader use of HZ vaccination in adult patients with IBD [31–35]. A recombinant zoster vaccine (RZV) is available for the prevention of HZ and is recommended by the Advisory Committee on Immunization Practices (ACIP) for immunocompetent adults aged ≥50 years [36]. More recently, the ACIP has also recommended the use of RZV in individuals aged ≥19 years who are or who will be immunodeficient or immunosuppressed because of disease or therapy [22]. Accompanying clinical guidance from the Centers for Disease Control and Prevention (CDC) states that in those patients with autoimmune or inflammatory conditions, RZV should be given prior to initiation of immunosuppressive medications if possible and ideally not during acute disease exacerbations or flares [37].

Recent observational studies in the USA evaluating RZV use in IBD patients have provided initial evidence of substantial reductions in HZ risk [38, 39]. In a study comparing the risk of HZ in IBD patients receiving RZV vs IBD patients not receiving RZV, Khan *et al*. reported that no HZ cases were observed in a total of 655 IBD patients aged 50–60 years after receiving a complete two-dose RZV schedule, while 69 HZ cases were observed in a total of 5,995 unvaccinated IBD patients (with a resultant HR for HZ of 0) [38]. For those patients aged >60 years, 8 cases of HZ were observed in a total of 4,220 fully vaccinated IBD patients and 268 cases in 20,554 unvaccinated IBD patients, with an HR for HZ of 0.39 (95% CI, 0.19–0.80) [38]. It should be recognized that relatively few person-years of follow-up were available in the fully vaccinated cohorts (715.8 PY in those aged 50–60 years and 4,444.9 PY in those aged >60 years) compared with the unvaccinated cohorts (17,560.7 and 58,633.1, respectively, for the same age strata) [38]. Safety data in IBD patients indicate that the frequency of local and systemic adverse reactions are comparable with those seen in broader immunocompetent patient populations with a low prevalence of IBD flares [40, 41]. In a recent US study that evaluated RZV safety in 1,677 IBD patients receiving it, the cumulative incidence of disease flares within 90 days was similar to that of a matched cohort of unvaccinated IBD patients (1.2% vs 1.0%) [41].

Previous studies were conducted prior to the recent recommendations for RZV use in younger immunodeficient or immunosuppressed patients [22]. Based on the increased incidence of HZ in patients with IBD compared with the general adult population, it will be important to consider approaches to prevention to avoid HZ disease burden in patients with IBD.

## Supplementary Material

goad016_Supplementary_Data

## Data Availability

GSK makes available anonymized individual participant data and associated documents from interventional clinical studies that evaluate medicines, upon approval of proposals submitted to www.clinicalstudydatarequest.com. To access data for other types of GSK sponsored research, for study documents without patient-level data, and for clinical studies not listed, please submit an enquiry via the website.
