# Supplementary material for: Incidence and risk of herpes zoster in patients with ulcerative colitis and Crohn’s disease in the USA
Source: Gastroenterol Rep (Oxf). 2023 Apr 12;11:goad016. doi: 10.1093/gastro/goad016 (PMC10097435; doi:10.1093/gastro/goad016)
Supplement: goad016_Supplementary_Data [file goad016_supplementary_data.pdf]

Supplementary Material

Incidence and risk of herpes zoster in patients with ulcerative colitis and Crohn's disease in the United States

Supplementary Table 1. Codes used to identify relevant medication use in study cohorts

Code type	Code	Description	Code type	Code	Description
**Aminosalicylic acid (5-ASA)**			**Methotrexate**		
GPI	09 00 00 10 00	Aminosalicylic acid	GPI	21 30 00 50 00	Methotrexate
GPI	52 50 00 30	Mesalamine	GPI	66 25 00 50 00	Methotrexate (antirheumatic)
GPI	52 50 00 40 10	Olsalazine	GPI	21 30 00 50 10	Methotrexate sodium
GPI	52 50 00 20 10	Balsalazide	GPI	66 25 00 50 10	Methotrexate sodium (antirheumatic)
GPI	52 50 00 60 00	Sulfasalazine	HCPCS	J8610	Methotrexate oral 2.5 mg
			HCPCS	J9250	Methotrexate sodium, 5 mg
			HCPCS	J9260	Methotrexate sodium, 50 mg
**Thiopurines**			**Biologics**		
GPI	21 30 00 40 00	Mercaptopurine	GPI	52 50 50 40	Infliximab
GPI	96 66 50 53 89	Mercaptopurine (bulk)	GPI	66 27 00 15	Adalimumab
GPI	96 66 50 53 90	Mercaptopurine monohydrate	GPI	66 27 00 40 00	Golimumab
GPI	99 40 60 10 00	Azathioprine	GPI	52 50 50 20 10	Certolizumab
GPI	99 40 60 10 10	Azathioprine sodium	GPI	52 50 30 80 00	Vedolizumab
GPI	21 30 00 60 00	Thioguanine	GPI	52 50 40 70 00	Ustekinumab (IV)
GPI	96 80 56 27 30	Thioguanine (bulk)	GPI	90 25 05 85 00	Ustekinumab
HCPCS	S0108	Mercaptopurine, oral, 50 mg	HCPCS	J1745	Injection, infliximab, excludes biosimilar, 10 mg
HCPCS	J7500	Azathioprine, oral, 50 mg	HCPCS	Q5102	Injection, infliximab, 10 mg
HCPCS	J7501	Azathioprine, parenteral, 100 mg	HCPCS	Q5103	Injection, infliximab-dyyb, biosimilar, (inflectra), 10 mg
**Janus kinase (JAK) inhibitors**			HCPCS	Q5104	Injection, infliximab-abda, biosimilar, (renflexis), 10 mg
GPI	66 60 30 65 10	Tofacitinib citrate	HCPCS	Q5109	Injection, infliximab-qbtx, biosimilar, (ixifi), 10 mg
GPI	96 80 70 20 10	Tofacitinib citrate (bulk)	HCPCS	Q5121	Injection, infliximab-axxq, biosimilar, (avsola), 10 mg
			HCPCS	J0135	Injection, adalimumab, 20 mg
			HCPCS	J1602	Injection, golimumab, 1 mg, for intravenous use
			HCPCS	J0717	Injection, certolizumab pegol, 1 mg
			HCPCS	J3380	Injection, vedolizumab, 1 mg
			HCPCS	C9026	Injection, vedolizumab, 1 mg (Deleted 2016-01-01)

			HCPCS	J3358	Ustekinumab, for intravenous injection, 1 mg
			HCPCS	C9487	Ustekinumab, for intravenous injection, 1 mg (Deleted 2017-07-01)
			HCPCS	Q9989	Ustekinumab, for intravenous injection, 1 mg (Deleted 2017-12-31)
			HCPCS	J3357	Ustekinumab, for subcutaneous injection, 1 mg
**Systemic steroids**			**Herpes zoster vaccination**		
GPI	22 10 00 30	Methylprednisolone	CPT	90736	Zoster (shingles) vaccine (HZV), live, for subcutaneous injection
GPI	22 10 00 40	Prednisolone	CPT	90750	Zoster (shingles) vaccine (HZV), recombinant, subunit, adjuvanted, for intramuscular use
GPI	22 10 00 45	Prednisone	HCPCS	G2160	Patient received at least one dose of the herpes zoster live vaccine or two doses of the herpes zoster recombinant vaccine (at least 28 days apart) anytime on or after the patient's 50th birthday before or during the measurement period
GPI	22 10 00 25	Hydrocortisone	HCPCS	G2161	Patient had prior adverse reaction caused by zoster vaccine or its components any time during or before the measurement period
GPI	96 56 88 11 30	Hydrocortisone hemisuccinate (bulk)	HCPCS	M1064	Shingrix vaccine documented as administered or previously received
GPI	22 10 00 12 00	Budesonide	GPI	17 10 00 95 10	Zoster vaccine live
GPI	96 44 82 12 00	Budesonide (bulk)	GPI	17 10 00 95 40	Zoster vaccine recombinant adjuvanted
GPI	89 15 00 07 00	Budesonide (intrarectal)	NDC	50090-3372-00	Shingrix, 1 KIT IN 1 KIT (50090-3372-0) * .5 ML IN 1 VIAL (58160-828-03) * .5 ML IN 1 VIAL (58160-829-03)
HCPCS	J1020	Injection, methylprednisolone acetate, 20 mg	NDC	50090-5147-00	Shingrix, 1 KIT IN 1 KIT (50090-5147-0) * .5 ML IN 1 VIAL (58160-828-01) * .5 ML IN 1 VIAL (58160-829-01)

HCPCS	J1030	Injection, methylprednisolone acetate, 40 mg	NDC	58160-828-01	Shingrix, 0.5 mL in 1 lyophilized antigen vial
HCPCS	J1040	Injection, methylprednisolone acetate, 80 mg	NDC	58160-829-01	Shingrix, 0.5 mL in 1 adjuvant suspension vial
HCPCS	J2920	Injection, methylprednisolone sodium succinate, up to 40 mg	NDC	58160-819-12	Shingrix, 1 KIT in 1 CARTON * .5 mL in 1 VIAL (58160-828-01) *.5 mL in 1 VIAL (58160-829-01)
HCPCS	J2930	Injection, methylprednisolone sodium succinate, up to 125 mg	NDC	58160-828-03	Shingrix, 0.5 mL in 1 lyophilized antigen vial
HCPCS	J7509	Methylprednisolone oral, per 4 mg	NDC	58160-829-03	Shingrix, 0.5 mL in 1 adjuvant suspension vial
HCPCS	J2650	Injection, prednisolone acetate, up to 1 ml	NDC	58160-823-11	Shingrix, 1 KIT in 1 CARTON * .5 mL in 1 VIAL (58160-828-03) * .5 mL in 1 VIAL (58160-829-03)
HCPCS	J7510	Prednisolone oral, per 5 mg	NDC	00006-4963-00	Zostavax, 1 VIAL, SINGLE-DOSE in 1 CARTON > .65 mL in 1 VIAL, SINGLE-DOSE
HCPCS	J7506	Prednisone, oral, per 5 mg	NDC	00006-4963-01	Zostavax
HCPCS	J7512	Prednisone, immediate release or delayed release, oral, 1 mg	NDC	00006-4963-41	Zostavax, 10 VIAL, SINGLE-DOSE in 1 CARTON > .65 mL in 1 VIAL, SINGLE-DOSE
HCPCS	J1700	Injection, hydrocortisone acetate, up to 25 mg			
HCPCS	J1710	Injection, hydrocortisone sodium phosphate, up to 50 mg			
HCPCS	J1720	Injection, hydrocortisone sodium succinate, up to 100 mg			

CPT, Current Procedural Terminology; HCPCS, Healthcare Common Procedure Coding System; GPI, Generic Product Identifier; IV, intravenous; NDC, National Drug Code.

Supplementary Table 2. Relevant baseline covariates included in propensity score

**Demographic and clinical characteristics included in propensity score**
Year of index date
Age at index date
Sex
Geographic region
Insurance plan type
CCI
Presence of any comorbidity potentially associated with HZ
Presence of any additional immunosuppressive condition
Markers of IBD severity (weight loss, malnutrition, anaemia)
IBD-related surgeries or procedures

Patient demographic characteristics were identified as of the index date. Clinical characteristics were assessed during the 6-month period immediately prior to the index date.

CCI, modified Charlson–Quan comorbidity index; HZ, herpes zoster; IBD, inflammatory bowel disease.

Supplementary Table 3. Baseline demographics and clinical characteristics of the study cohorts

	UC (*N* = 29,928)	Non-IBD (*N* = 11,839,329)	Standardised difference[a]	CD (*N* = 25,959)	Non-IBD (*N* = 11,839,329)	Standardised difference[a]
**CCI conditions, *n* (%)**						
Chronic pulmonary disease	4,227 (14.1)	937,016 (7.9)	19.8%	3,853 (14.8)	937,016 (7.9)	21.8%
Peripheral vascular disease	2,575 (8.6)	505,132 (4.3)	17.7%	1,905 (7.3)	505,132 (4.3)	13.1%
Moderate or severe renal disease	2,379 (7.9)	521,550 (4.4)	14.7%	2,107 (8.1)	521,550 (4.4)	15.3%
Diabetes (type 1 or 2) with end-organ damage	1,972 (6.6)	521,914 (4.4)	9.6%	1,459 (5.6)	521,914 (4.4)	5.6%
Diabetes (type 1 or 2) without end-organ damage	2,923 (9.8)	1,004,696 (8.5)	4.4%	2,281 (8.8)	1,004,696 (8.5)	1.1%
Heart failure	1,603 (5.4)	337,571 (2.9)	12.6%	1,253 (4.8)	337,571 (2.9)	10.3%
Cerebrovascular disease	1,448 (4.8)	333,675 (2.8)	10.5%	1,144 (4.4)	333,675 (2.8)	8.5%
Rheumatologic disease	1,269 (4.2)	173,685 (1.5)	16.7%	1,212 (4.7)	173,685 (1.5)	18.6%
Any malignant tumour	1,942 (6.5)	409,720 (3.5)	13.9%	1,447 (5.6)	409,720 (3.5)	10.2%
Mild liver disease	1,472 (4.9)	191,914 (1.6)	18.5%	1,493 (5.8)	191,914 (1.6)	21.9%
Myocardial infarction	777 (2.6)	138,213 (1.2)	10.5%	583 (2.2)	138,213 (1.2)	8.3%
Peptic ulcer disease	403 (1.3)	39,473 (0.3)	11.1%	421 (1.6)	39,473 (0.3)	13.1%
Dementia	555 (1.9)	150,096 (1.3)	4.7%	375 (1.4)	150,096 (1.3)	1.5%
Metastatic solid tumour	273 (0.9)	41,453 (0.4)	7.1%	186 (0.7)	41,453 (0.4)	5.0%
Hemiplegia	171 (0.6)	34,760 (0.3)	4.2%	127 (0.5)	34,760 (0.3)	3.1%
Moderate or severe liver disease	144 (0.5)	12,532 (0.1)	6.9%	107 (0.4)	12,532 (0.1)	6.0%
AIDS	81 (0.3)	20,931 (0.2)	2.0%	42 (0.2)	20,931 (0.2)	0.4%
**Comorbidities potentially associated with HZ, *n* (%)**	2,048 (6.8)	317,280 (2.7)	19.6%	2,097 (8.1)	317,280 (2.7)	23.9%
Sicca syndrome (Sjögren)	147 (0.5)	20,281 (0.2)	5.6%	126 (0.5)	20,281 (0.2)	5.5%
Other interstitial lung disease	272 (0.9)	42,017 (0.4)	7.0%	209 (0.8)	42,017 (0.4)	5.9%
Systemic lupus erythematosus	190 (0.6)	25,165 (0.2)	6.5%	197 (0.8)	25,165 (0.2)	7.9%
Psoriasis	465 (1.6)	80,398 (0.7)	8.3%	591 (2.3)	80,398 (0.7)	13.2%
Psoriatic arthritis	146 (0.5)	18,139 (0.2)	5.9%	176 (0.7)	18,139 (0.2)	8.2%
Ankylosing spondylitis	151 (0.5)	7715 (0.1)	8.2%	245 (0.9)	7,715 (0.1)	12.4%
Sarcoidosis	65 (0.2)	11,883 (0.1)	2.9%	61 (0.2)	11,883 (0.1)	3.3%
Giant cell arteritis	37 (0.1)	4,381 (0.0)	3.1%	20 (0.1)	4,381 (0.0)	1.7%
Idiopathic pulmonary fibrosis	24 (0.1)	3,727 (0.0)	2.1%	12 (0.0)	3,727 (0.0)	0.7%

Multiple sclerosis	135 (0.5)	30,734 (0.3)	3.2%	129 (0.5)	30,734 (0.3)	3.9%
Wegener's granulomatosis	10 (0.0)	1,378 (0.0)	1.5%	6 (0.0)	1,378 (0.0)	0.9%
Scleroderma	9 (0.0)	1,581 (0.0)	1.1%	4 (0.0)	1,581 (0.0)	0.2%
Dermato/polymyositis	1 (0.0)	43 (0.0)	0.7%	0 (0.0)	43 (0.0)	0.3%
Rheumatoid arthritis	828 (2.8)	118,232 (1.0)	13.0%	814 (3.1)	118,232 (1.0)	15.0%
Polymyalgia rheumatica	0 (0.0)	0 (0.0)	0.0%	0 (0.0)	0 (0.0)	0.0%
**Additional immunosuppressive conditions, *n* (%)**	3,161 (10.6)	216,181 (1.8)	36.2%	4,354 (16.8)	216,181 (1.8)	51.5%
Use of chemotherapy for solid and haematological malignancies in prior 6 months	2,876 (9.6)	174,009 (1.5)	35.6%	4,185 (16.1)	174,009 (1.5)	51.7%
Solid organ transplant	223 (0.7)	21,360 (0.2)	8.3%	132 (0.5)	21,360 (0.2)	5.6%
Symptomatic HIV	81 (0.3)	20,989 (0.2)	2.0%	42 (0.2)	20,989 (0.2)	0.4%
Haematopoietic stem-cell transplant	18 (0.1)	1,561 (0.0)	2.5%	11 (0.0)	1,561 (0.0)	1.8%
**Markers of IBD severity prior to index date, *n* (%)**	3,821 (12.8)	471,588 (4.0)	31.7%	3,730 (14.4)	471,588 (4.0)	36.0%
Weight loss	906 (3.0)	82,728 (0.7)	17.2%	924 (3.6)	82,728 (0.7)	19.8%
Malnutrition	352 (1.2)	16,006 (0.1)	12.9%	381 (1.5)	16,006 (0.1)	14.9%
Anaemia	3,086 (10.3)	396,204 (3.3)	27.6%	2,958 (11.4)	396,204 (3.3)	30.8%
**Patients with any all-cause healthcare costs, *n* (%)**	29,506 (98.6)	9,394,748 (79.4)	61.4%	25,581 (98.5)	9,394,748 (79.4)	61.2%
Total healthcare costs, PPPY, 2020 USD, mean ± SD, (median)	$21,436 ± 52,404 ($6,054)	$6,024 ± 25,387 ($818)	37.4%	$27,229 ± 57,766 ($10,035)	$6,024 ± 25,387 ($818)	47.5%

Patient demographic characteristics were identified as of the index date. Clinical characteristics were assessed during the 6-month period strictly prior to the index date.
[a] Standardised differences of 20%, 50%, and 80% suggest small, medium and large differences between cohorts, respectively [1].
AIDS, acquired immunodeficiency syndrome; CCI, modified Charlson–Quan comorbidity index; CD, Crohn's disease; HIV, human immunodeficiency virus; HZ, herpes zoster; IBD, inflammatory bowel disease; *N*, number of patients in the cohort; *n*, number of patients in a category; PPPY, per-person-per-year; SD, standard deviation; UC, ulcerative colitis.

Supplementary Figure 1. Histogram of propensity scores for the UC, CD, and non-IBD cohorts

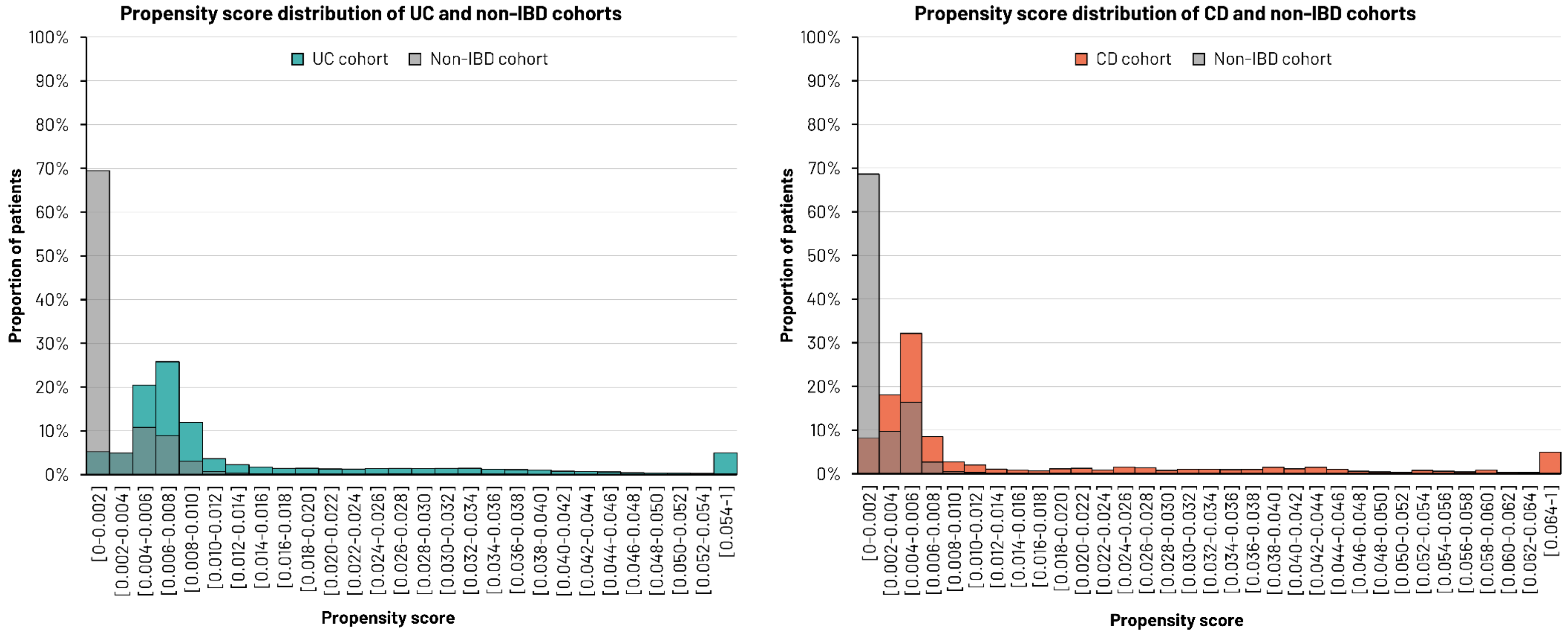

Propensity scores were estimated using logistic regression with IBD status as the outcome (i.e. UC vs non-IBD; CD vs non-IBD). Predictors in the propensity score models included key baseline demographics (year of index date, age, sex, geographic region, and insurance type) measured as of the index date and clinical characteristics (CCI scores, any comorbidity potentially associated with HZ, additional immunosuppressive conditions, markers of IBD severity, and IBD-related surgeries or procedures) assessed during the 6-month period strictly prior to the index date. CCI, modified Charlson–Quan comorbidity index; CD, Crohn's disease; HZ, herpes zoster; IBD, inflammatory bowel disease; UC, ulcerative colitis.

Supplementary Figure 2. IBD-related medication use in UC and CD cohorts at baseline

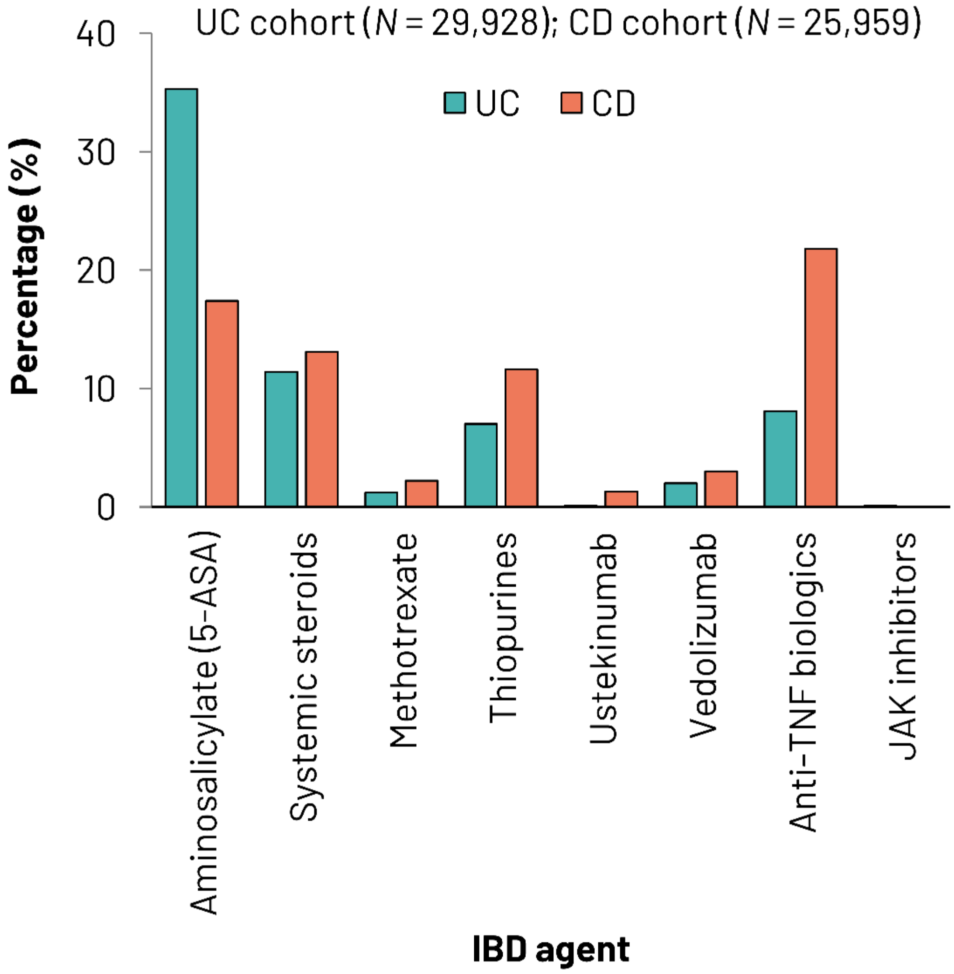

CD, Crohn’s disease; IBD, inflammatory bowel disease; JAK, Janus kinase; *N*, number of patients in the cohort; TNF, tumour necrosis factor; UC, ulcerative colitis.

Supplementary Table 4. Crude (unadjusted) and adjusted incidence rate ratios for HZ in UC and CD cohorts compared with the non-IBD cohort by age group

	UC (*N* = 29,928)	Non-IBD (*N* = 11,839,329)				CD (*N* = 25,959)	Non-IBD (*N* = 11,839,329)			
	**Incidence rate per 1,000 person-years**[a]		Unadjusted IRR (95% CI)	**Adjusted IRR (95% CI)**[b]	***P*-value**[c]	**Incidence rate per 1,000 person-years**[a]		Unadjusted IRR (95% CI)	**Adjusted IRR (95% CI)**[b]	***P*-value**[c]
**Overall**	13.64	7.95	1.72 (1.60–1.83)	1.35 (1.26–1.44)	<0.001	15.94	7.95	2.01 (1.88–2.14)	1.66 (1.56–1.77)	<0.001
18–49 years	9.10	3.99	2.28 (1.96–2.65)	1.70 (1.46–1.99)	<0.001	12.41	3.99	3.11 (2.76–3.51)	2.26 (1.99–2.56)	<0.001
18–29 years	5.43	2.11	2.57 (1.69–3.91)	1.85 (1.18–2.89)	0.007	8.68	2.11	4.11 (3.12–5.41)	3.35 (2.48–4.52)	<0.001
30–39 years	9.47	4.19	2.26 (1.77–2.89)	1.80 (1.40–2.31)	<0.001	12.83	4.19	3.06 (2.49–3.77)	2.29 (1.84–2.85)	<0.001
40–49 years	10.65	5.53	1.92 (1.55–2.38)	1.58 (1.27–1.97)	<0.001	14.70	5.53	2.66 (2.22–3.17)	2.00 (1.67–2.41)	<0.001
50–64 years	13.93	8.71	1.60 (1.39–1.84)	1.40 (1.21–1.61)	<0.001	15.80	8.71	1.81 (1.59–2.07)	1.47 (1.29–1.68)	<0.001
≥65 years	16.32	11.79	1.38 (1.27–1.51)	1.23 (1.13–1.34)	<0.001	19.45	11.79	1.65 (1.50–1.81)	1.44 (1.31–1.59)	<0.001

[a] Incidence rates calculated as the number of patients with incident HZ divided by the person-time observed expressed per 1,000 person-years.
[b] Adjusted incidence rate ratios (aIRRs) were calculated using generalised linear models with a Poisson distribution, adjusting for patients' propensity score and relevant baseline characteristics/baseline covariates.
[c] *P*-values for aIRRs were calculated using the Poisson distribution.
aIRR, adjusted incidence rate ratio; CD, Crohn's disease; CI, confidence interval; HZ, herpes zoster; IBD, inflammatory bowel disease; *N*, number of patients in the cohort; SD, standard deviation; UC, ulcerative colitis.

Supplementary Table 5. Crude (unadjusted) and adjusted incidence rate ratios for HZ in UC and CD cohorts compared with the non-IBD cohort for those receiving IBD-related medications

	**UC (*N* = 29,928)**	**Non-IBD[a] (*N* = 11,839 329)**				**CD (*N* = 25,959)**	**Non-IBD[a] (*N* = 11,839,329)**			
	**Incidence rate per 1,000 person-years[b]**		Unadjusted IRR (95% CI)	**Adjusted IRR (95% CI)[c]**	***P*-value[d]**	**Incidence rate per 1,000 person-years[b]**		Unadjusted IRR (95% CI)	**Adjusted IRR (95% CI)[c]**	***P*-value[d]**
**IBD-medication use**										
No therapy, 5-ASA or budesonide	12.17	7.46	1.63 (1.50–1.77)	1.24 (1.14–1.35)	<0.001	12.79	7.46	1.72 (1.56–1.89)	1.31 (1.19–1.44)	<0.001
Thiopurine or methotrexate alone	18.50	16.65	1.11 (0.85–1.46)	1.82 (1.31–2.54)	<0.001	28.20	16.65	1.69 (1.38–2.08)	2.51 (1.94–3.25)	<0.001
Vedolizumab or ustekinumab alone	9.74	8.43	1.16 (0.72–1.85)	1.87 (0.80–4.40)	0.149	11.78	8.43	1.40 (0.99–1.97)	1.16 (0.62–2.17)	0.651
Adalimumab, certolizumab or infliximab alone	14.39	13.87	1.04 (0.79–1.36)	1.67 (1.14–2.45)	0.009	15.34	13.87	1.11 (0.92–1.33)	1.75 (1.32–2.32)	<0.001
Any biologic in combination with methotrexate or thiopurine	15.75	19.03	0.83 (0.48–1.42)	1.09 (0.45–2.62)	0.853	22.80	19.03	1.20 (0.85–1.69)	1.90 (1.06–3.40)	0.032
JAK inhibitors	11.99	22.10	0.54 (0.20–1.46)	0.37 (0.10–1.41)	0.145	18.17	22.10	0.82 (0.12–5.87)	0.81 (0.06–11.10)	0.875
Corticosteroid other than budesonide, alone or in combination with the medications above	24.07	20.69	1.16 (0.98–1.38)	1.10 (0.93–1.31)	0.270	28.64	20.69	1.38 (1.19–1.61)	1.36 (1.16–1.59)	<0.001

[a] Comparisons are between UC and CD cohorts versus patients without IBD receiving the same medications, presumably for other autoimmune or inflammatory conditions.
[b] Incidence rates calculated as the number of patients with incident HZ divided by the person-time observed expressed per 1,000 person-years.
[c] Adjusted incidence rate ratios (aIRRs) were calculated using generalised linear models with a Poisson distribution, adjusting for patients' propensity score and relevant baseline characteristics/baseline covariates.
[d] *P*-values for aIRRs were calculated using the Poisson distribution.
5-ASA; aminosalicylate; aIRR, adjusted incidence rate ratio; CD, Crohn's disease; CI, confidence interval; HZ, herpes zoster; IBD, inflammatory bowel disease; JAK, Janus kinase; *N*, number of patients in the cohort; SD, standard deviation; UC, ulcerative colitis.

Trademark:
Shingrix is a trademark owned by or licensed to GSK.
Zostavax is a trademark of Merck & Co., Inc.

References for supplementary material:

1. Cohen J. *Statistical power analysis for the behavioral sciences*. Mahwah: Lawrence Erlbaum Associates, 1988.
